# A novel cytochrome P450, CYP3201B1, is involved in (*R*)‐mandelonitrile biosynthesis in a cyanogenic millipede

**DOI:** 10.1002/2211-5463.12170

**Published:** 2017-02-01

**Authors:** Takuya Yamaguchi, Yasumasa Kuwahara, Yasuhisa Asano

**Affiliations:** ^1^ Biotechnology Research Center and Department of BiotechnologyToyama Prefectural UniversityImizuJapan; ^2^ JSTERATOAsano Active Enzyme Molecule ProjectImizuJapan

**Keywords:** *Chamberlinius hualienensis*, cyanogenesis, cytochrome P450, hydroxynitrile, millipede

## Abstract

Specialized arthropods and more than 2500 plant species biosynthesize hydroxynitriles and release hydrogen cyanide as a defensive mechanism. The millipede *Chamberlinius hualienensis* accumulates (*R*)‐mandelonitrile as a cyanide precursor. Although biosynthesis of hydroxynitriles in cyanogenic plants and in an insect are extensively studied, (*R*)‐mandelonitrile biosynthesis in cyanogenic millipedes has remained unclear. In this study, we identified the biosynthetic precursors of (*R*)‐mandelonitrile and an enzyme involved in (*R*)‐mandelonitrile biosynthesis. Using deuterium‐labelled compounds, we revealed that (*E*/*Z*)‐phenylacetaldoxime and phenylacetonitrile are the biosynthetic precursors of (*R*)‐mandelonitrile in the millipede as well as other cyanogenic organisms. To identify the enzymes involved in (*R*)‐mandelonitrile biosynthesis, 50 cDNAs encoding cytochrome P450s were cloned and coexpressed with yeast cytochrome P450 reductase in yeast, as cytochrome P450s are involved in the biosynthesis of hydroxynitriles in other cyanogenic organisms. Among the 50 cytochrome P450s from the millipede, CYP3201B1 produced (*R*)‐mandelonitrile from phenylacetonitrile but not from (*E*/*Z*)‐phenylacetaldoxime, whereas plant and insect cytochrome P450s catalysed the dehydration of aldoximes and hydroxylation of nitriles. CYP3201B1 is not phylogenetically related to cytochrome P450s from other cyanogenic organisms, indicating that hydroxynitrile biosynthetic cytochrome P450s have independently evolved in distant species. Our study will shed light on the evolution of cyanogenesis among plants, insects and millipedes.

**Database:**

Nucleotide sequence data are available in the DDBJ/EMBL/GenBank databases under the accession numbers LC125356–LC125405.

**Abbreviations**
BA: benzaldehyde
BzCN: benzoyl cyanide
CNglcs: cyanogenic glycosides
CYP: cytochrome P450
HCN: hydrogen cyanide
HCT‐TK: high‐capacity ion‐trap mass spectrometer
HNL: hydroxynitrile lyase
MAN: mandelonitrile
MN‐Ba: mandelonitrile benzoate
MOX: mandelonitrile oxidase
Nr: non‐redundant protein database
PAN: phenylacetonitrile
PAOx: phenylacetaldoxime
UPLC: ultra‐high‐performance liquid chromatography

Some specialized arthropods and more than 2500 species of plants are known to release hydrogen cyanide (HCN) 1. As HCN inhibits mitochondrial cytochrome *c*‐oxidase in the cellular respiration system 2, the compound plays a crucial role in their defense against enemies. Cyanogenesis has been well documented in plants and insects. Cyanogenic plants accumulate glycosides of hydroxynitriles called cyanogenic glycosides (CNglcs) as stable cyanide precursors 3. When plant tissues containing CNglcs are disrupted, CNglcs are degraded by β‐glucosidase and hydroxynitrile lyase (HNL) to release aldehydes or ketones and HCN via hydroxynitriles 4, 5. CNglcs are biosynthesized from amino acids through aldoximes, nitriles and hydroxynitriles with the aid of two cytochrome P450s (CYP79s and CYP71s) and UDP‐glucosyltransferases 6. Cyanogenic insects, such as *Zygaena filipendulae* L. (Lepidoptera) and *Paropsis atomaria* Olivier (Coleoptera), also biosynthesize and accumulate CNglcs as cyanide precursors 7, 8. *Zygaena filipendulae* utilizes two cytochrome P450s (CYP405A2 and CYP332A3) and a UDP‐glucosyltransferase to produce CNglcs 9, similarly as cyanogenic plants. Although the cytochrome P450s and UDP‐glucosyltranseferases of cyanogenic plants and *Z. filipendulae* are not phylogenetically related, these enzymes catalyse the same reactions.

Cyanogenesis in millipedes has been known for more than 130 years 10. Polydesmid millipedes examined to date are generally cyanogenic 11, 12, 13. These millipedes accumulate mandelonitrile (MAN) as a cyanide precursor and eject cyanogenic secretions through small openings called ozopores on the dorsal surface near the tips of some of the paired notal projections 14. In the polydesmid millipedes, *Oxidus gracilis* (C. L. Koch) and *Harpaphe haydeniana* (Wood), MAN is biosynthesized from l‐phenylalanine (l‐Phe) 15, 16. However, (*R*)‐MAN biosynthesis in cyanogenic millipedes has remained unclear 17.

The polydesmid millipede *Chamberlinius hualienensis* Wang is invaded from Taiwan to Japan 18. An outbreak of *C. hualienensis* in Japan was first recorded in 1983, and this millipede has been expanding its habitat in Japan 19, 20. Large swarms of this millipede enter houses and sometimes cause train delays 19. The millipede releases (*R*)‐MAN, benzaldehyde (BA), benzoyl cyanide (BzCN), mandelonitrile benzoate (MN‐Ba) and HCN as defensive chemicals 21. Recently, two enzymes involved in cyanogenesis have been purified from several kilograms of *C. hualienensis* and characterized. One enzyme is HNL localized in the reaction chamber of defensive glands 22. When the millipede is alarmed, (*R*)‐MAN stored in the reservoir of defensive glands is mixed with HNL and degraded to BA and HCN, which are secreted externally through ozopores. The other is mandelonitrile oxidase (MOX) localized in lymph 23. This enzyme catalyses the conversion of (*R*)‐MAN into BzCN, which is spontaneously condensed with (*R*)‐MAN to produce MN‐Ba and HCN 24. When their bodies are disrupted by predators, MOX and its substrate (*R*)‐MAN likely come into contact. *C. hualienensis* likely biosynthesizes (*R*)‐MAN from l‐Phe similarly as other cyanogenic millipedes 15, 16. However, the biosynthetic intermediates and biosynthetic enzymes of (*R*)‐MAN in *C. hualienensis* as well as other cyanogenic millipedes have not been elucidated, although (*R*)‐MAN is the key compound of cyanogenesis in the millipede.

In this study, we determined that (*E*/*Z*)‐phenylacetaldoxime (PAOx) and phenylacetonitrile (PAN) are the biosynthetic precursors of (*R*)‐MAN in *C. hualienensis*. We cloned cDNAs encoding cytochrome P450s from *C. hualienensis* based on its transcriptome data and constructed a functional library of cytochrome P450s in which 44 isoforms are coexpressed with yeast cytochrome P450 reductase in *Saccharomyces cerevisiae*. Further, we identified a cytochrome P450 catalysing the last step of (*R*)‐MAN biosynthesis in *C. hualienensis* through functional screening from the library.

## Results

### (*E*/*Z*)‐PAOx and PAN are the biosynthetic precursors of (*R*)‐MAN, which is unlikely to be converted to CNglcs in *Chamberlinius hualienensis*

**Figure 1:**
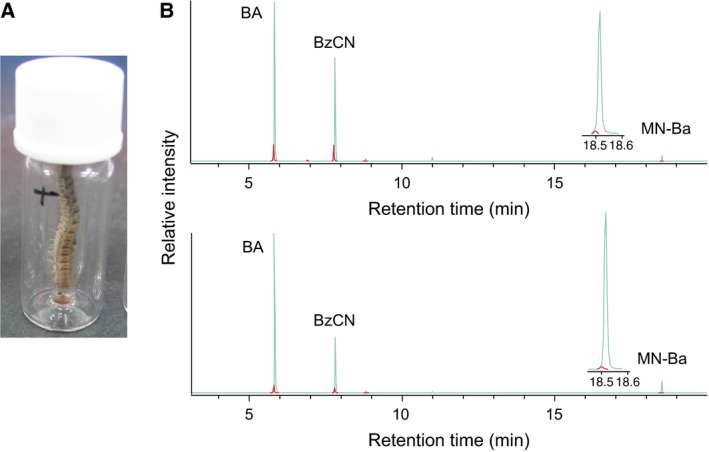
Incorporation of deuterium‐labelled compounds into mandelonitrile (MAN)‐derived compounds. (A) ‘Force feeding’ of deuterium‐labelled compounds to *Chamberlinius hualienensis*. (B) Typical gas chromatograms of defensive compounds extracted from the millipedes fed D_5_‐(*E*/*Z*)‐phenylacetaldoxime (upper chromatogram) or D_5_‐phenylacetonitrile (lower chromatogram). Benzaldehyde (BA), benzoyl cyanide (BzCN) and mandelonitrile benzoate (MN‐Ba) were monitored at their base peak ion, *m*/*z* 105 (green line). Penta‐deuterated defensive compounds were monitored at *m*/*z* 110 (red line).

As aldoximes and nitriles are the biosynthetic precursors of hydroxynitriles in cyanogenic plants and insects 9, 25, 26, 27, D_5_‐(*E*/*Z*)‐PAOx and D_5_‐PAN were synthesized and applied to *C. hualienensis* via the ‘force feeding’ method (Fig. 1A). The incorporation of deuterium‐labelled compounds into MAN, BA, BzCN and MN‐Ba was analysed using a gas chromatography–mass spectrometry (GC–MS) system. However, we could not directly detect the incorporation of deuterium‐labelled compounds into MAN, as MAN is decomposed to BA and HCN in the injection port of a GC–MS system 25. On the contrary, the incorporation of deuterium‐labelled compounds into BA, BzCN and MN‐Ba was detected when each labelled compound was fed to millipedes. Because their base peak ion is commonly *m*/*z* 105, natural and deuterated compounds could be monitored at *m*/*z* 105 and *m*/*z* 110, respectively (Fig. 1B). The incorporation rates of deuterium‐labelled compounds into natural compounds were approximately 2.5–8%. Considering that BA, BzCN and MN‐Ba are derived from (*R*)‐MAN 22, 23, 24, (*E*/*Z*)‐PAOx and PAN are the biosynthetic precursors of (*R*)‐MAN in *C. hualienensis*.

We also searched for CNglcs in the millipede, as hydroxynitriles are converted to CNglcs as a storage form in cyanogenic plants and insects 6, 9. (*R*)‐MAN‐derived glucosides, prunasin and amygdalin were analysed using a liquid chromatography–mass spectrometry (LC–MS) system. None of the compounds was detected in *C. hualienensis*, whereas prunasin and amygdalin were detected in the kernel of the cyanogenic plant *Prunus mume* Sieb. et Zucc. (Fig. S1), as previously reported 28. These results were in agreement with that for the glycosylated form of MAN, which was not detected in the cyanogenic millipede *H. haydeniana* 16, suggesting that glycosylation of (*R*)‐MAN is unlikely to occur in *C. hualienensis*.

### *Chamberlinius hualienensis* has unique cytochrome P450s

**Figure 2:**
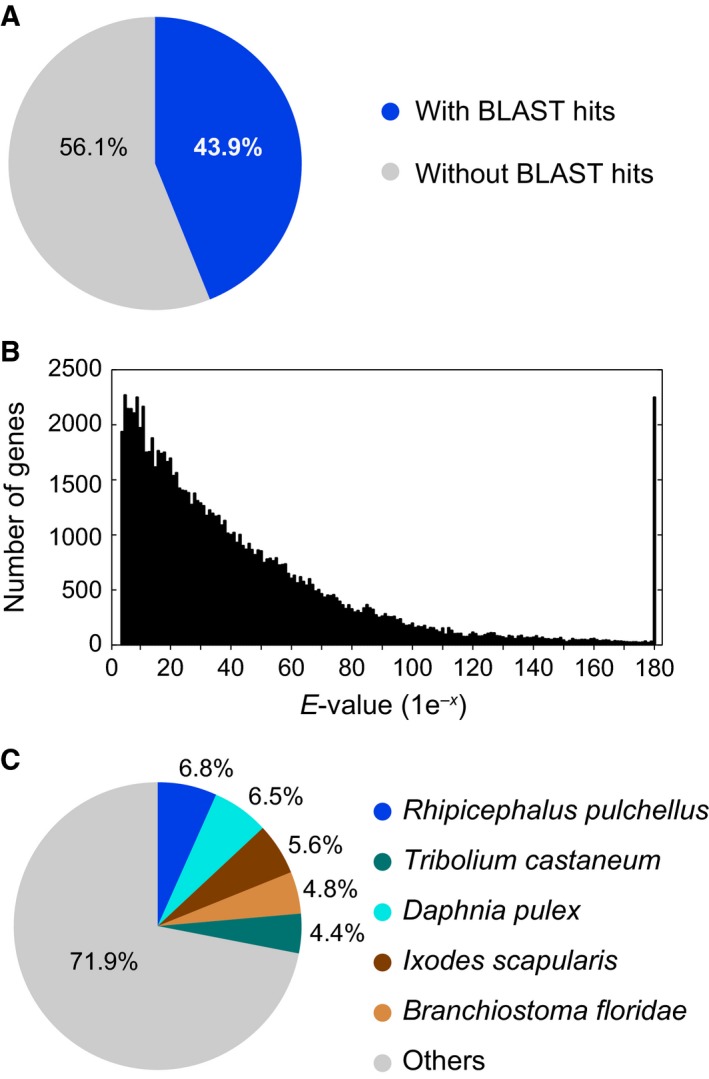
Summary of blastx results for the transcriptomic sequences vs. GenBank non‐redundant protein database. (A) Proportions of *Chamberlinius hualienensis* transcriptomic sequences with or without blastx hits. (B) *E*‐value distribution of blastx hits for matched *C. hualienensis* transcriptomic sequences using an *E*‐value cut‐off of 1.0 × 10^−3^. (C) Taxonomic distribution of the top blastx hits of *C. hualienensis* transcriptomic sequences.

To identify candidate (*R*)‐MAN biosynthetic enzymes, we first performed a TBLASTN search 29 against *C. hualienensis* transcriptome data to elucidate transcriptomic sequences having more than 40% identity at the amino acid level with CYP79D16, CYP71AN24, CYP405A2 or CYP332A3, which are involved in hydroxynitrile biosynthesis in the cyanogenic plant *P. mume* and the cyanogenic insect *Z. filipendulae* 9, 25. However, we failed to identify sequences having similarity with those cytochrome P450s. Therefore, we further analysed the transcriptome data of *C. hualienensis* to obtain cytochrome P450s. For the contigs and singletons, we performed homology searches against the non‐redundant protein database (Nr) of the NCBI using the blastx 30. Approximately 43.9% of sequences displayed significant similarity to protein in the Nr (Fig. 2A). The majority of matches ranged from 1e^−100^ to 1e^−4^ (Fig. 2B). The translated transcriptomic sequences of *C. hualienensis* tended to have similarities with those of genome‐sequenced arthropods but no strong relation was observed with specific species (Fig. 2C).

**Figure 3:**
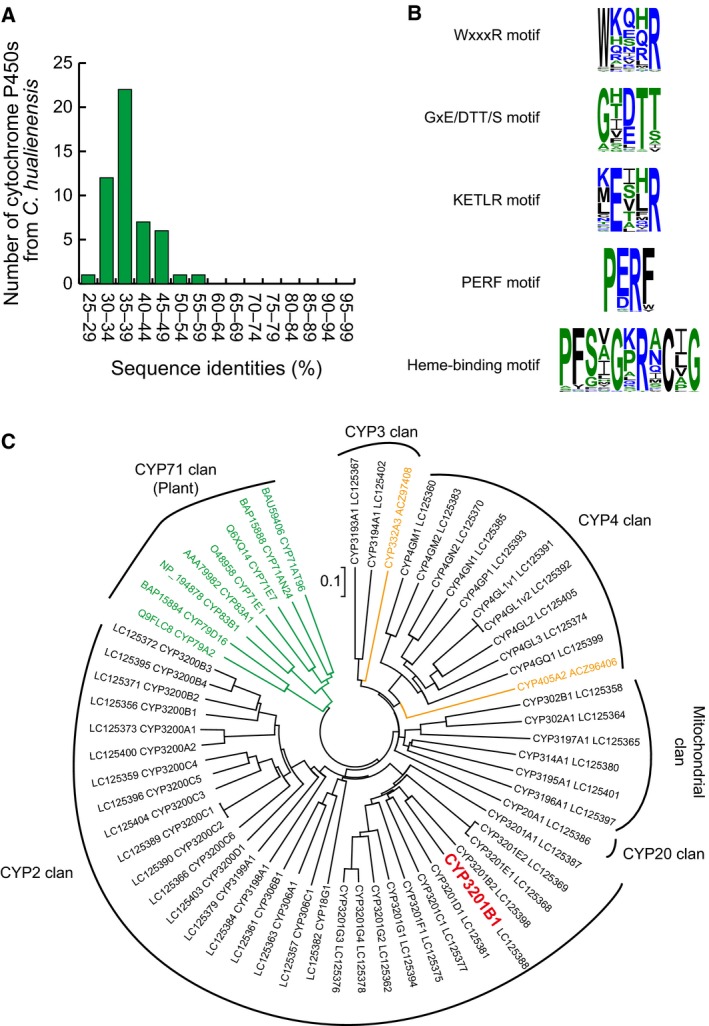
Sequence and phylogenetic analysis of cytochrome P450s from *Chamberlinius hualienensis* and other cyanogenic organisms. (A) Distribution of sequence identities between cytochrome P450s from *C. hualienensis* and cytochrome P450s in the GenBank non‐redundant protein database. (B) Conserved sequences of cytochrome P450s from *C. hualienensis*. Sequence logos of conserved sequences in cytochrome P450s from *C. hualienensis* were created using WebLogo (http://weblogo.threeplusone.com). (C) Phylogenetic analysis of cytochrome P450s from *C. hualienensis* and those from plants and insects involved in cyanogenesis. The phylogenetic tree was constructed by the neighbour‐joining method with a 1000‐replicate bootstrap test. Cytochrome P450s from *C. hualienensis*, plants and *Zygaena filipendulae* are shown in black, green and orange, respectively. CYP3201B1 is highlighted in red.

Through the annotation processes, 47 contigs and 70 singletons were annotated as cytochrome P450s. Because partial sequences were combined after 5′‐ and 3′‐RACE, we could determine 50 cDNA sequences encoding cytochrome P450s, which were responsible for 100 of 117 contigs and singletons. Cytochrome P450s from *C. hualienensis* had low amino acid sequence identities with previously reported cytochrome P450s and the majority of their sequence identities were ranged from 30% to 49% (Fig. 3A). In general, CYP names are assigned by amino acid sequence identity, and cytochrome P450s with more than 40% and 55% identities are placed in the same family and subfamily, respectively 31. Accordingly, 33 and 10 cytochrome P450s from *C. hualienensis* were assigned to novel families and novel subfamilies within existing families, respectively (Table S1; D. R. Nelson, P450 Nomenclature Committee).

All cytochrome P450s from *C. hualienensis* had conserved sequences of cytochrome P450s (WxxxR motif, GxE/DTT/S motif, KETLR motif, PERF motif and heme‐binding motif) (Fig. 3B). As these motifs correspond to the conserved tertiary structure and enzyme functions of cytochrome P450s 32, these cytochrome P450s likely catalyse hydroxylation of their substrates, similarly as other cytochrome P450s.

Phylogenetic analysis did not uncover close relationships of cytochrome P450s from *C. hualienensis* with those from cyanogenic plants and an insect (Fig. 3C). Cytochrome P450s from *C. hualienensis* were clustered into five groups corresponding to CYP2, CYP3, CYP4, CYP20 and mitochondrial clan (Fig. 3C). Because the mitochondrial cytochrome P450s of arthropods are clearly linked to the ecdysteroid metabolism pathway 33, we reasoned that cytochrome P450s belong to the CYP2, CYP3, CYP4 or CYP20 clans are likely involved in (*R*)‐MAN biosynthesis in *C. hualienensis*. The entire list of CYP names, CYP clans and accession numbers of cytochrome P450s from *C. hualienensis* are summarized in Table S1.

### CYP3201B1 catalyses the stereoselective hydroxylation of PAN into (*R*)‐MAN

**Figure 4:**
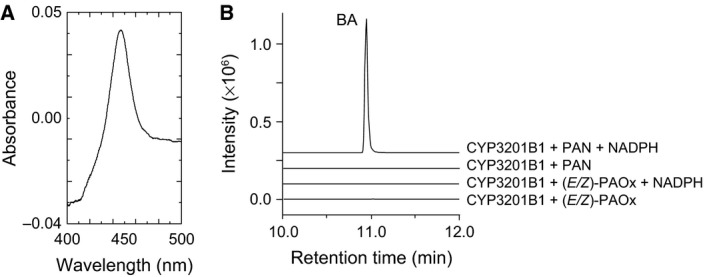
Heterologous expression of CYP3201B1 and identification of reaction products produced from (*E*/*Z*)‐phenylacetaldoxime (PAOx) or phenylacetonitrile (PAN) by CYP3201B1. (A) Carbon monoxide difference spectra of microsomes harbouring CYP3201B1. (B) Microsomes harbouring CYP3201B1 were incubated with PAN or (*E*/*Z*)‐PAOx in the presence or absence of NADPH. The decomposed product of mandelonitrile, benzaldehyde, was monitored at *m*/*z* 106.

To identify cytochrome P450s involved in (*R*)‐MAN biosynthesis, whole‐cell activity assays were performed using yeast transformants and l‐Phe, (*E*/*Z*)‐PAOx or PAN as the substrate because whole‐cell activity assays can detect the catalytic activities of cytochrome P450s even though their expression level is below the detection limit of the CO difference spectrum assay 34. Forty‐four cytochrome P450s belonging to the CYP2, CYP3, CYP4 and CYP20 clans were coexpressed with yeast NADPH‐cytochrome P450 reductase in *S. cerevisiae* and used for whole‐cell activity assays. Among the transformants, a transformant carrying a CYP3201B1 expression plasmid consumed PAN. To characterize CYP3201B1, microsomes harbouring CYP3201B1 were prepared, and CYP3201B1 expression was analysed using CO difference spectroscopy. The microsome harbouring CYP3201B1 displayed the characteristic Soret peak of cytochrome P450 (Fig. 4A), indicating that CYP3201B1 was produced in a correctly folded and active form. The microsome harbouring CYP3201B1 produced BA from PAN in the presence of NADPH but not from (*E*/*Z*)‐PAOx (Fig. 4B), indicating that CYP3201B1 converts PAN into MAN, which is decomposed to BA and HCN in the injection port of a GC–MS system 25.

**Figure 5:**
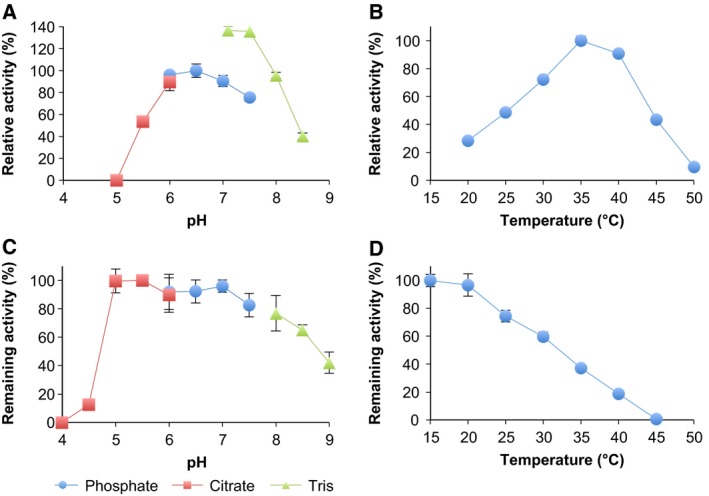
Effects of pH and temperature on the catalysis of CYP3201B1. (A) Optimum pH. The reaction was performed at 30 °C for 5 min in the following buffers: 50 mm sodium citrate buffer (pH 5.0–6.0); 50 mm potassium phosphate buffer (KPB, pH 6.0–7.5) and 50 mm Tris‐HCl buffer (pH 7.1–8.5) (B) Optimum temperature. The reactions were performed in 50 mm KPB (pH 6.5) at various temperatures (20–50 °C) for 5 min. (C) pH stability. Remaining activity was measured after microsomes harbouring CYP3201B1 were incubated at 25 °C for 15 min at in the pH range of 4.0–9.0 without substrate. (D) Thermal stability. Remaining activity was measured after microsomes harbouring CYP3201B1 were incubated at 15–45 °C for 60 min without substrate. Enzyme activity was calculated using the values of mandelonitrile and benzaldehyde. Values are the means ± SD; *n* = 4.

**Figure 6:**
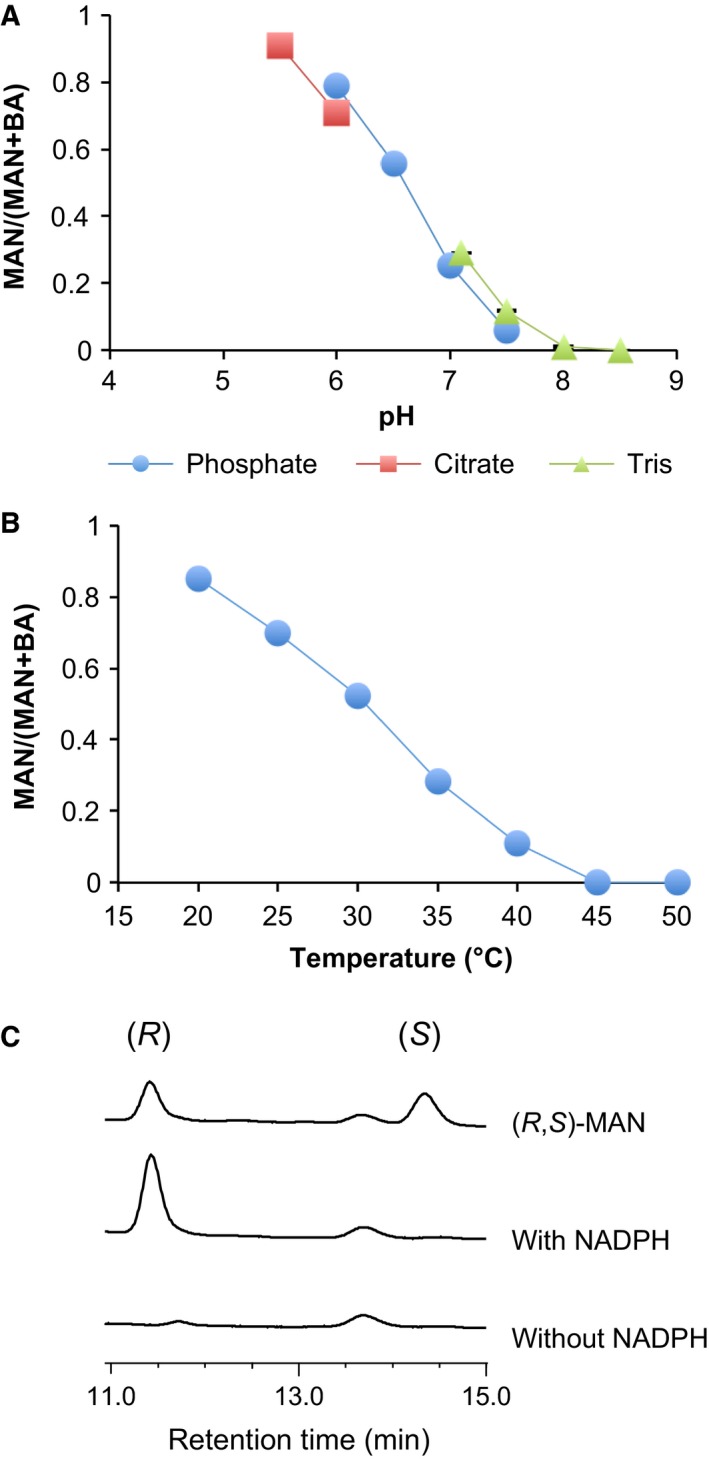
Chirality assignment of mandelonitrile (MAN) converted from phenylacetonitrile (PAN) by CYP3201B1. (A) Ratio of MAN to benzaldehyde (BA) converted from PAN in various buffers. (B) Ratio of MAN to BA converted from PAN at various temperatures. (C) Chirality assignment of MAN. Microsomes harbouring CYP3201B1 were incubated with PAN in the presence or absence of NADPH at pH 6.0 and 20 °C for 10 min. MAN converted from PAN was analysed using an HPLC system equipped with a chiral column. Values are the means ± SD;* n* = 4.

Because *C. hualienensis* accumulates (*R*)‐MAN but not (*S*)‐MAN 23, CYP3201B1 was thought to catalyse the stereoselective hydroxylation of PAN. However, an assignment of the stereochemistry of hydroxynitriles produced by hydroxynitrile‐forming cytochrome P450s from plants and an insect has not been reported, as their enzyme activities were evaluated via the detection of HCN or aldehydes as reaction products due to the instability of hydroxynitriles 9, 25, 26, 27. It has been reported that MAN is relatively stable at low pH and low temperature 35. Thus, to detect MAN directly as a reaction product, the effects of pH and temperature on the catalysis of CYP3201B1 and stability of MAN converted from PAN were elucidated. A microsomal system harbouring CYP3201B1 had an optimal pH and temperature of 6.5 and 35 °C (Fig. 5A,B), respectively. The enzymes were stable in the range of pH 5–7 and incubation at 20 °C for 15 min (Fig. 5C,D). MAN could be detected using an ultra‐high‐performance liquid chromatography (UPLC) system at a pH and temperature of less than 7.5 and 45 °C, respectively, and degradation of MAN was almost prevented at pH 6.0 and 20 °C (Fig. 6A,B). Therefore, we performed an assay with CYP3201B1 in the presence or absence of NADPH at pH 6.0 and 20 °C, and reaction products were analysed using an HPLC system equipped with a chiral column. As expected, (*R*)‐MAN, but not (*S*)‐MAN, was detected in the presence of NADPH (Fig. 6C), indicating that CYP3201B1 catalysed the stereoselective hydroxylation of PAN into (*R*)‐MAN.

### CYP3201B1 has a relatively narrow substrate specificity and high catalytic efficiency

The substrate specificity of CYP3201B1 was examined using structurally related compounds of PAN, as listed in Table 1. CYP3201B1 acted on PAN as well as 4‐hydroxyPAN, 2‐methylPAN and 3‐methylPAN, whereas did not act on 3‐phenylpropionitrile, indole‐3‐acetonitrile, 3‐phenyl‐1‐propyne or 3‐phenyl‐1‐propene (Table 1). The substrate specificity of CYP3201B1 is relatively narrow compared to that of plant hydroxynitrile‐forming CYP71s 25, 26, 36.

**Table 1:** Kinetic parameters of CYP3201B1. ND, not determined

Substrate	Structure	*K*_m_ (μm)	*k*_cat_ (min^−1^)	*k*_cat_/*K*_m_
PAN	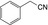	9.2 ± 2.3	184.5 ± 12.9	20.1
4‐HydroxyPAN	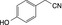	15.8 ± 1.2	42.4 ± 0.7	2.7
2‐MethylPAN	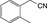	6.6 ± 1.6	67.4 ± 4.2	10.2
3‐MethylPAN	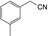	6.9 ± 1.3	65.4 ± 2.6	9.5
3‐Phenylpropionitrile	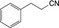	ND	ND	ND
Indole‐3‐acetonitrile	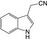	ND	ND	ND
3‐Phenyl‐1‐propyne	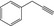	ND	ND	ND
3‐Phenyl‐1‐propene	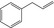	ND	ND	ND

CYP3201B1 exhibited a *K*_m_ of 9.2 μm and *k*_cat_ of 184.5 min^−1^ for PAN (Table 1). These low *K*_m_ and *k*_cat_ values were similar to those for CYP71s from cyanogenic plants 25, 27, 36. The catalytic efficiency (*k*_cat_/*K*_m_) of 20.1 min^−1^·μm ^−1^ for PAN was the highest among all tested substrates, indicating that CYP3201B1 specifically reacts with PAN in *C. hualienensis*.

## Discussion

(*R*)‐MAN is the key compound of cyanogenic millipedes in the release of HCN. Recently, HNL and MOX, which are responsible for cyanogenesis in the polydesmid millipede *C. hualienensis*, were identified and characterized 22, 23. However, the (*R*)‐MAN biosynthetic pathway of *C. hualienensis* and the enzymes involved in the pathway have not been reported. To identify the biosynthetic precursors of (*R*)‐MAN, deuterium‐labelled compounds were synthesized and administered to *C. hualienensis*. Next, we obtained 50 cDNAs encoding cytochrome P450s from *C. hualienensis* on the basis of transcriptome data and performed a functional screening of cytochrome P450s to identify the enzymes involved in the biosynthesis of (*R*)‐MAN. In this study, we identified (*E*/*Z*)‐PAOx and PAN as the biosynthetic precursors of (*R*)‐MAN. Furthermore, we identified a cytochrome P450, CYP3201B1 catalysing the stereoselective hydroxylation of PAN into (*R*)‐MAN but not the dehydration of (*E*/*Z*)‐PAOx into PAN.

Clans have been proposed as a higher order for grouping cytochrome P450s, defining groups of CYP families that consistently cluster together on phylogenetic trees 31. In *C. hualienensis*, CYP2, CYP3, CYP4, CYP20 and mitochondrial clans were found (Fig. 3C). CYP3201B1 and more than half of cytochrome P450s from *C. hualienensis* belong to the CYP2 clan, whereas CYP3 or CYP4 clans are major clans in genome‐sequenced insect species such as *Drosophila melanogaster*,* Tribolium castaneum*,* Anopheles gambiae* and *Bombyx mori* 37, 38. CYP405A2 and CYP332A3 from *Z. filipendulae* belong to the CYP4 clan and CY3 clan, respectively 9. CYP79s and CYP71s from cyanogenic plants belong to the CYP71 clan, which is the largest clan in plants 39. Thus, cytochrome P450s involved in the biosynthesis of hydroxynitriles belong to different clans among a millipede, a cyanogenic insect and cyanogenic plants. Cytochrome P450s within a clan likely diverged from a common ancestor gene 40, suggesting that cytochrome P450s involved in hydroxynitrile biosynthesis have independently evolved in distant species.

**Figure 7:**
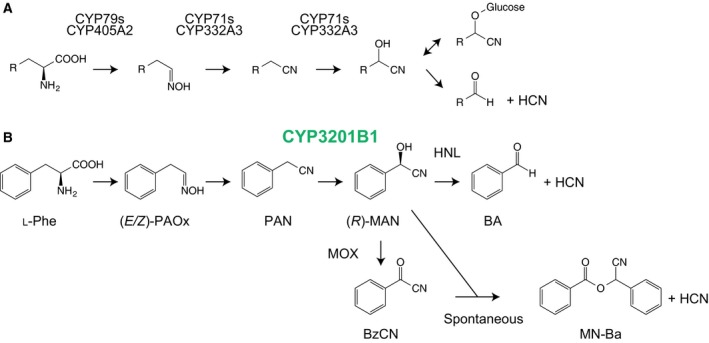
Cyanogenesis pathway of cyanogenic plants and *Zygaena filipendulae* (A) and proposed cyanogenesis pathway of *Chamberlinius hualienensis* (B). (A) Amino acids are converted into hydroxynitriles via aldoximes and nitriles by two cytochrome P450s. Hydroxynitriles are glycosylated to cyanogenic glycosides as a storage form. Cyanogenic glycosides are degraded to aldehydes or ketones and hydrogen cyanide (HCN) by the combination of β‐glucosidase and hydroxynitrile lyase (HNL). (B) l‐Phe is converted into (*R*)‐mandelonitrile (MAN) via (*E*/*Z*)‐phenylacetaldoxime (PAOx) and phenylacetonitrile (PAN). The conversion of PAN into (*R*)‐MAN is catalysed by CYP3201B1. HCN is produced from (*R*)‐MAN via two pathways. One is the degradation of (*R*)‐MAN to benzaldehyde and HCN by HNL. The other is the conversion of (*R*)‐MAN into benzoyl cyanide (BzCN) by MOX. BzCN is spontaneously condensed with (*R*)‐MAN to form mandelonitrile benzoate (MN‐Ba) and HCN.

In cyanogenic plants and an insect, two multifunctional cytochrome P450s catalyse the conversion of amino acids into hydroxynitriles (Fig. 7A). First, cytochrome P450 catalyses the conversion of amino acids into aldoximes, which are further converted to hydroxynitriles via nitriles by a second cytochrome P450 6. In our observations, (*E*/*Z*)‐PAOx and PAN were converted into MAN‐derived compounds (Fig. 1), indicating that the biosynthetic precursors of hydroxynitriles are conserved among cyanogenic organisms. However, CYP3201B1 identified in this study acted on PAN but not (*E*/*Z*)‐PAOx (Fig. 4), in contrast to the hydroxynitrile‐forming cytochrome P450s of cyanogenic plants and an insect. Thus, dehydration of (*E*/*Z*)‐PAOx is likely catalysed by another enzyme(s) in the millipede. CYP71AT96 from the giant knotweed, *Fallopia sachalinensis* catalyses the conversion of (*E*/*Z*)‐PAOx into PAN but not to MAN 41 in herbivore‐induced PAN biosynthesis 42, 43. Dehydration of aldoximes to nitriles is also catalysed by the bacterial enzyme aldoxime dehydratase 44, 45, 46, 47. Dehydration of (*E*/*Z*)‐PAOx to PAN in *C. hualienensis* is presumably catalysed by such enzymes with aldoxime dehydratase activity. Although the biosynthetic precursors of hydroxynitriles are conserved among millipedes, an insect and plants, different sets of enzymes are likely involved in the biosynthesis of hydroxynitriles in *C. hualienensis* and other cyanogenic organisms.

CYP3201B1 effectively catalysed the stereoselective conversion of PAN into (*R*)‐MAN with a *K*_m_ of 9.2 ± 2.3 μm and *k*_cat_ of 184.5 ± 12.9 min^−1^ (Table 1). In cyanogenic plants, the biosynthesis of CNglcs is highly channelled to avoid the release of toxic intermediates into the cytosol 48, 49. Therefore, the low *K*_m_ value and high *k*_cat_ of CYP3201B1 promote the efficient metabolism of PAN in the same manner as CYP71s from cyanogenic plants. In cyanogenic plants and insects, hydroxynitriles are further converted to CNglcs as a storage form with the aid of UDP‐glucosyltransferases 9, 50, 51, 52. Knocking out of *UGT85B1*, which encodes the enzyme catalysing the glucosylation of 4‐hydroxyMAN into dhurrin, causes the self‐cyanide intoxication of *Sorghum bicolor* (L.) Moench 53. However, cyanogenic millipedes including *C. hualienensis* are unlikely to convert (*R*)‐MAN into CNglcs (Fig. S1) 16. The pH of the defensive secretion of *C. hualienensis* and other cyanogenic millipedes is maintained at approximately 4.0 13, 23, 54. In our observation, (*R*)‐MAN was stable at pH 5.5 (Fig. 5E). The low pH condition of defensive glands likely enabled storage of (*R*)‐MAN without degradation. Taken together, we propose the (*R*)‐MAN biosynthesis and cyanogenesis pathway of *C. hualienensis* as depicted in Fig. 7B. Our study will help to reveal the (*R*)‐MAN biosynthesis in cyanogenic millipedes and shed light on the evolution of cyanogenesis among millipedes, insects and plants.

## Materials and methods

### Animals and plants

*Chamberlinius hualienensis* was collected in Kagoshima prefecture, Japan. Millipedes were reared with the litter of the Japanese cedar *Cryptomeria japonica* D. Don or steamed potatoes at room temperature until use. The younger stadia were obtained from eggs laid by the millipedes. Fruits of the Japanese apricot *P. mume* were purchased from local markets in Japan.

### Chemicals

(*E*/*Z*)‐PAOx was synthesized as described previously 44. Deuterium‐labelled *2,3,4,5,6‐*D_5_‐(*E*/*Z*)‐PAOx and *2,3,4,5,6‐*D_5_‐PAN were synthesized starting from *2,3,4,5,6‐*D_5_‐bromobenzene (Cambridge Isotope Laboratories, Inc., Andover, MA, USA) via the Grignard reaction, oxidation, aldoxime formation and dehydration, as follows; D_5_‐phenylmagnesium bromide prepared from D_5_‐bromobenzene was reacted with ethylene oxide to give D_5_‐2‐phenylethanol. The alcohol was oxidized to D_5_‐2‐phenylacetaldehyde and then lead to D_5_‐(*E*/*Z*)‐PAOx by a reaction with 50% aq. NH_2_OH. D_5_‐PAN was obtained by dissolving D_5_‐(*E*/*Z)*‐PAOx in acetic anhydrate. All other chemicals were purchased from commercial suppliers.

### Application of deuterium‐labelled compounds and analysis of defensive compounds

To elucidate the (*R*)‐MAN biosynthetic pathway of *C. hualienensis*, deuterium‐labelled compounds were administered to millipedes by ‘force feeding’. Fifth stadium of *C. hualienensis* were each separately placed in a microvolume insert containing 4–5 μL of 1000 ppm aq. D_5_‐(*E*/*Z*)‐PAOx or D_5_‐PAN with their heads down, as shown in Fig. 1A. After force feeding at room temperature for 16 h, the defensive compounds were extracted after the addition of *n*‐hexane (80–100 μL) for 3 min, and 4 μL of the extract were analysed using a GC–MS (7890A GC System coupled with a 5975C inert XL EI/CI MSD with a Triple‐Axis Detector operated at 70 eV; Agilent Technologies, Santa Clara, CA, USA) system equipped with an HP‐5 ms capillary column (0.25 mm i.d. × 30 m, 0.25 μm film thickness; Agilent Technologies) according to a previous report 55.

### Detection of CNglcs

To detect l‐Phe‐derived CNglcs, prunasin and amygdalin, 20 g of millipedes or 200 mg of the kernel of *P. mume* were disrupted under liquid N_2_. Millipede or plant powder was mixed with 80 mL or 400 μL of ice‐cold methanol (MeOH) respectively. The MeOH extract from millipedes was concentrated under N_2_. The extract from *P. mume* was diluted with water up to fivefold. Extracts from *C. hualienensis* and *P. mume* were analysed using an LC–MS system equipped with a COSMOSIL 5C_18_‐MS‐II Packed Column (150 mm × 2.0 mm i.d.; Nacalai Tesuque, Kyoto, Japan) under the following conditions: mobile phase A, 0.1% formic acid in water; and mobile phase B, 0.1% formic acid in acetonitrile and 5–50% linear gradient of B for 25 min and 100% linear gradient of B for 5 min delivered at 0.2 mL·min^−1^. MS was simultaneously performed in the positive‐ion mode using a high‐capacity ion‐trap mass spectrometer (HCT‐TK; Bruker Daltonics, Billerica, MA, USA) via electrospray ionization. Prunasin and amygdalin were monitored with *m*/*z* 318 [M + Na]^+^ and *m*/*z* 475 [M + NH_4_]^+^, respectively.

### Bioinformatics

Assembled sequences of our previously sequenced transcriptome data of *C. hualienensis* (DNA Databank of JAPAN Sequence Read Archive: DRA003259) were annotated using blastx 30 to search the Nr of the NCBI, and the *E*‐value threshold was 1.0 × 10^−3^.

### Cloning of cDNAs encoding cytochrome P450s from *C. hualienensis*

Total RNA was prepared from the degutted body of *C. hualienensis* using the TRIzol (Thermo Fisher Scientific, Waltham, MA, USA). cDNA was synthesized using the GeneRacer Kit (Thermo Fisher Scientific) according to the manufacturer's protocol. Oligonucleotide primers for 5′‐ and 3′‐RACE were designed based on assembled sequences. PCR was performed using the Tks Gflex DNA Polymerase (Takara, Shiga, Japan) or the TaKaRa Ex Taq (Takara). RACE products were cloned into the pCR‐Blunt (Thermo Fisher Scientific) or the pCRII vector (Thermo Fisher Scientific) to determine sequences. Coding sequences of cytochrome P450s were amplified by PCR and cloned into the pCR‐Blunt or pCRII vector. At least four individual clones were sequenced to avoid errors derived from PCR. Oligonucletide primers used in this study are summarized in Table S2. Sequence data were submitted to the DDBJ/EMBL/GenBank databases under the accession numbers LC125356–LC125405 (Table S1).

### Identification of cytochrome P450s involved in (*R*)‐MAN biosynthesis

To identify cytochrome P450s involved in (*R*)‐MAN biosynthesis, cytochrome P450s and yeast cytochrome P450 reductase were coexpressed in *S. cerevisiae*. The coding sequences of cytochrome P450s were reamplified by PCR using appropriate primer sets (Table S2) and cloned into the *Hin*dIII site of the pGYR vector 56 using the In‐Fusion HD Cloning kit (Clontech Laboratories, Palo Alto, CA, USA). After confirmation of the insert DNA sequences, expression vectors were transformed into *S. cerevisiae* AH22 57 by the lithium acetate method 58. Transformants were inoculated into 1 mL of a SD‐His medium and incubated at 30 °C for 3 days. Cultured medium (20 μL) was transferred into 2 mL of the concentrated SD‐His medium and cultured at 30 °C for 2 days. Cells were harvested by centrifugation (1500 ***g***, 5 min, 4 °C) and suspended in 0.2 mL of whole‐cell assay buffer (100 mm potassium phosphate buffer [KPB]; pH 7.4), 7 mm magnesium acetate, 10 mm glucose). The reaction was started by the addition of 10 mm l‐Phe, 1 mm (*E*/*Z*)‐PAOx or 1 mm PAN. After incubation at 30 °C for 180 min, the reactions were terminated by adding 200 μL of ice‐cold 0.2% formic acid in 50% acetonitrile. Resulting insoluble materials were precipitated by centrifugation (1500 ***g***, 15 min, 4 °C). Supernatant was recovered and filtered using the MultiScreen HTS HV filter plate (Merck Millipore, Billerica, MA, USA). The reaction products in filtrate were analysed using a Nexera UPLC (Shimadzu, Kyoto, Japan) system equipped with a COSMOCIL 2.5C_18_‐MS‐II column (75 mm × 2.0 mm i.d.; Nacalai Tesque) under the following conditions: column oven temperature of 40 °C; solvent, 0.1% formic acid in 25% acetonitrile; and flow rate of 0.4 mL·min^−1^. (*E*/*Z*)‐PAOx, PAN and MAN were monitored according to the absorbance at 210 nm. BA was monitored at 248 nm.

### Preparation of microsomes

To characterize CYP3201B1, microsomes harbouring CYP3201B1 and yeast NADPH‐cytochrome P450 reductase were prepared as described previously 25. In brief, the yeast transformants cultured with the concentrated SD‐His medium were harvested and disrupted using a Multi‐beads Shocker (Yasui Kikai, Osaka, Japan). Cell debris and beads were discarded by centrifugation (10 000 ***g***, 10 min, 4 °C). Supernatant was collected and ultra‐centrifuged (150 000 ***g***, 60 min, 2 °C) to precipitate microsomes harbouring CYP3201B1. The amount of CYP3201B1 was quantified via a carbon monoxide (CO) difference spectrum assay 59 using an extinction coefficient of ε_450–490_ = 91 mm ^−1^·cm^−1^ 60.

### Assay of PAN hydroxylase activity

A 200‐μL reaction mixture containing 50 nm CYP3201B1, 50 mm KPB (pH 6.5), 2 mm NADPH and 1 mm PAN was incubated at 35 °C for 5 min. The reaction was started by the addition of NADPH. Reaction products were extracted using 400 μL of *n*‐hexane and analysed using a GC–MS–QP2010 Plus (Shimadzu) equipped with a TC‐70 column (i.d., 60 m × 0.25 mm; film thickness, 0.25 μm; GL Science, Tokyo, Japan). Helium was used as the carrier gas and was applied at 30 mL·min^−1^. The column temperature was increased by 10 °C·min^−1^ from 80 °C to 250 °C as described previously 25.

### Chirality assignment of MAN

A 400‐μL reaction mixture containing 500 nm of CYP3201B1, 50 mm KPB (pH 6.0), 2 mm NADPH, and 1 mm PAN was incubated at 20 °C for 10 min. The reaction was started by the addition of NADPH. Reaction products were extracted using 600 μL of *n*‐hexane:2‐propanol (85 : 15) and analysed using a Prominence UFLC Liquid Chromatography System (Shimadzu) equipped with a CHIRALCEL OJ‐H column (250 mm × 4.6 mm i.d.; Daicel Corporation, Tokyo, Japan) under the following conditions: Column oven temperature of 30 °C, solvent of *n*‐hexane:2‐propanol (85 : 15) and flow rate of 1.0 mL·min^−1^.

### Substrate specificity of CYP3201B1

The substrate specificity of CYP3201B1 was assayed under the standard assay condition using 1 mm 4‐hydroxyPAN, 2‐methylPAN, 3‐methylPAN, 3‐phenylpropionitrile, indole‐3‐acetonitrile, 3‐phenyl‐1‐propyne and 3‐phenyl‐1‐propene. The reaction products were extracted and analysed using a GC–MS system equipped with a TC‐70 column as described in Section ‘Assay of PAN hydroxylase activity’.

### Measurement of the kinetic parameters

To determine *K*_m_ and *k*_cat_, the reactions were conducted under the standard assay condition using 2.5–200 μm PAN, 2–500 μm 4‐hydroxyPAN, 2–100 μm 2‐methylPAN and 2–200 μm 3‐methylPAN. The reactions were terminated by the addition of 200 μL of 50% acetonitrile in water containing 0.2% formic acid. As hydroxynitriles are spontaneously decomposed to aldehydes and HCN, the sum of both compounds was quantified as the reaction products using an UPLC system. *K*_m_ and *k*_cat_ were determined by curve fitting of the data with kaleidagraph (version 4.1.2; Synergy Software, Reading, PA, USA) using the Michaelis–Menten equation.

### Phylogenetic analysis

Phylogenetic analysis was performed using the cytochrome P450s from *C. hualienensis* and previously reported cytochrome P450s involved in nitrile biosynthesis, which were obtained from the GenBank database. Multiple sequence alignment of cytochrome P450s was performed using the clustalx program 61 with default parameters. The phylogenetic tree was constructed by the neighbour‐joining method using mega 6 62. The significance level for the phylogenetic tree was assessed by bootstrap testing with 1000 replications.

## Author contributions

TY collected millipedes, carried out the molecular lab work, participated in data analysis, carried out sequence alignments, participated in the design of the study and drafted the manuscript; YK collected millipedes, carried out the chemical synthesis and tracing experiments, participated in the design of the study, and drafted the manuscript; YA collected millipedes, participated in data analysis, conceived of the study, designed the study, coordinated the study and drafted the manuscript. All authors gave final approval for publication.

## Supporting information


**Fig. S1.** Detection of prunasin and amygdalin in *Chamberlinius hualienensis* and *Prunus mume*.

**Table S1.** Classification of cytochrome P450s from *Chamberlinius hualienensis*.

**Table S2.** Oligonucleotide primers.
